# Role of interleukin 6 as a predictive factor for a severe course of Covid-19: retrospective data analysis of patients from a long-term care facility during Covid-19 outbreak

**DOI:** 10.1186/s12879-021-05945-8

**Published:** 2021-03-29

**Authors:** P. Sabaka, A. Koščálová, I. Straka, J. Hodosy, R. Lipták, B. Kmotorková, M. Kachlíková, A. Kušnírová

**Affiliations:** 1 Department of Infectology and Geographical Medicine, Faculty of Medicine, Comenius University in Bratislava, Bratislava, Slovakia; 2 Department of Infectology and Geographical Medicine, Faculty of Medicine, Slovak Medical University, Bratislava, Slovakia; 3 2nd Department of Neurology, Faculty of Medicine, Comenius University in Bratislava, Bratislava, Slovakia; 4 Emergency Department, University Hospital in Bratislava, Bratislava, Slovakia; 5 Institute of Molecular Biomedicine, Faculty of Medicine, Comenius University in Bratislava, Bratislava, Slovakia; 6 Institute of Physiology, Faculty of Medicine, Comenius University in Bratislava, Bratislava, Slovakia

**Keywords:** Covid-19, Long-term care facility, Interleukin 6, Hypoxemia

## Abstract

**Background:**

Covid-19 is a disease with high morbidity and mortality among elderly residents of long-term care facilities (LTCF). During an outbreak of SARS-CoV-2 infection in the LTCF an effective screening tool is essential to identify the patients at risk for severe disease. We explored the role of interleukin 6 (IL-6) as a predictor for severe disease during the outbreak of Covid-19 in one LTCF in Slovakia.

**Methods:**

We conducted a retrospective data analysis of cases of COVID-19, diagnosed during the outbreak in one LTCF in Slovakia between April 11, 2020, and May 5, 2020. Within 24 h after the diagnosis of Covid-19, clinical and laboratory screening was performed in the LTCF to identify patients in need of hospitalization. Patients with oxygen saturation below 90% were immediately referred to the hospital. Patients staying in the LFTC were monitored daily and those that developed hypoxemia were transferred to the hospital. We analyzed the association between the IL-6 at the initial assessment and development of hypoxemia during follow up and determined the cut-off of the IL-6 able to predict the development of hypoxemia requiring oxygen therapy.

**Results:**

Fifty-three patients (11 men, 42 women) with diagnosed Covid-19 were included in the analysis. 19 (53%) patients developed hypoxemia during the disease. Patients with hypoxemia had significantly higher concentrations of IL-6, C-reactive protein, procalcitonin, fibrinogen, total bilirubin, aspartate aminotransferase and alanine aminotransferase at initial screening. ROC analyses identified IL-6 as the most robust predictor of hypoxemia. The concentration of IL-6 > 24 pg/mL predicted the development of hypoxemia with the sensitivity of 100% and specificity of 88.9%. The positive and negative predictive values were 76.9, and 100% respectively.

**Conclusions:**

The concentration of IL-6 > 24 pg/mL at initial assessment predicted the development of hypoxemia requiring hospitalization with excellent sensitivity and good specificity. IL-6 appears as a potential predictor for the development of the severe Covid-19 and might serve for early identification of patients in need of hospitalization. Further studies are needed to evaluate the robustness of the use of IL-6 as an effective screening tool for the severe course of Covid-19.

**Supplementary Information:**

The online version contains supplementary material available at 10.1186/s12879-021-05945-8.

## Background

Coronavirus disease 2019 (Covid-19) is a disease caused by the zoonotic novel Coronavirus called SARS-CoV-2 [1]. Covid-19 quickly spread across the globe from a place of its origin in Hubei China and pandemic was declared by WHO on March 11, 2020 [2]. Most patients with Covid-19 experience mild self-limiting disease. However, up to 20% of known cases of Covid-19 are complicated by severe pneumonia which might result in acute respiratory distress syndrome (ARDS) which causes acute hypoxemic respiratory failure [1, 3]. The overall infection fatality ratio is estimated to be below 1% [4]. It should be noted that fatal outcome may occur at any age including children and young adults [5]. Also, physiologic condition, pregnancy is associated with higher risk of severe disease [6]. However, the risk of death in Covid-19 is increasing with age and a presence of comorbidities, especially cardiovascular diseases, diabetes mellitus and obesity [3]. According to data from the early China epidemic, the case-fatality ratio in the patients over 80 is up to 15% [7]. Elderly and highly comorbid residents of long-term care facilities (LTCF) are at high risk of Covid-19 associated morbidity and mortality [8–10]. In one well-documented outbreak in LTCF in Washington, USA, more than half of the infected residents required hospitalization and more than one third died [10]. On the background of the ongoing pandemic, the spreading of Covid-19 in LTCF might significantly burden the local health care system and markedly contributes to mortality. Timely and effective intervention is essential to reduce morbidity and mortality during the Covid-19 outbreak in the LTCF. Such intervention consists of quick identification of cases, immediate introduction of infection control measures, initial triage, and daily monitoring of patients. An effective screening tool is essential to identify the patients at risk for severe illness and death [11]. Such patients need close monitoring and early transfer to the hospital. Various biomarkers, especially inflammatory markers like C-reactive protein (CRP), ferritin, fibrinogen, D-dimer and Interleukin 6 (IL-6) are associated with Covid-19 progression [12, 13]. According to known evidence, IL-6 is superior to CRP and other markers of inflammation in predicting respiratory failure in Covid-19 [14, 15]. IL-6 appears to be the most important driver of immune dysregulation and ARDS in Covid-19 [16–19]. The role of systematic measurement of IL-6, CRP, and other markers of inflammation at the initial assessment during Covid-19 outbreak in LTCF and its ability to predict the severe course of disease is yet to be determined.

## Methods

We conducted a retrospective data analysis of laboratory-confirmed cases of COVID-19, diagnosed during the outbreak in one LTCF in Slovakia between April 11, 2020, and May 5, 2020. A confirmed case of COVID-19 was defined as a positive result on real-time reverse-transcriptase–polymerase-chain-reaction assay of nasopharyngeal swab specimens for SARS-CoV-2. In our analysis, we aimed to identify laboratory markers predicting the severe course of the disease at the initial assessment.

### Intervention

Within 24 h after the diagnosis of COVID-19, clinical and laboratory screening was performed in the LTCF by trained clinicians to identify patients in need of hospitalization. The clinical initial assessment consisted of measurement of vital signs (blood pressure, heart rate respiratory rate) and measurement of oxygen saturation by pulse oximetry. Additionally, the venous blood was drawn to measure concentrations of serum glucose, creatinine, urea, sodium, potassium, bilirubin, alanine aminotransferase (ALT), aspartate aminotransferase (AST), CRP, D-dimer, IL-6 and complete blood count. The blood samples were immediately transported to laboratory. The results were obtained and evaluated from 4 to 6 h after last blood drawn. After an initial assessment, daily monitoring consisting of measurement of vital signs and measurement of oxygen saturation by pulse oximetry was performed by the same clinician. The development of hypoxemia with oxygen saturation below 90% was considered as the criteria for severe disease with the need for hospitalization. Patients with oxygen saturation below 90% at the initial screening or during the daily monitoring were immediately referred to the hospital.

### Biochemical analysis

Concentrations of serum glucose, creatinine, urea, sodium, potassium, bilirubin, ALT, AST, were measured using spectrophotometry (Cobas Integra 400, Roche Diagnostics, Rotkreuz, Switzerland). CRP and D-dimer were measured using immunoturbidimetry (Cobas Integra 400, Roche Diagnostics, Rotkreuz, Switzerland). Serum IL-6 concentrations were measured using an immunoassay (Elecsys, Roche Diagnostics, Rotkreuz, Switzerland).

### Statistical analysis

Quantitative variables are expressed as medians and 25th and 75th percentiles. Data in our cohort according to the Kolmogorov-Smirnov test were non-parametric. Medians of quantitative variables between groups were compared using the Mann-Whitney nonparametric test. Effect sizes were assessed using Cohen’s d. Receiver operating characteristic curve (ROC) analysis was used to compare markers of hypoxemia. Only the biomarkers with different medians between groups were evaluated in ROC analysis. Optimal cut off values were also determined using ROC analysis. Sensitivity was calculated as the number of true positive divided by true positive + false negative. Specificity was calculated as the number of true negative divided by true negative + false positive. Association of baseline serum concentration of IL-6 > 24 pg/mL with the probability of development of hypoxemia requiring oxygen therapy (HRO) was assessed using multivariate logistic regression. For statistical analysis, SPSS version 26 (International Business Machines Corporation, Armonk, NY, USA) was used.

### Ethics

This study was carried out in concordance with The Code of Ethics of the World Medical Association (Declaration of Helsinki) for experiments involving humans and was approved by the local Ethical Committee of University Hospital Bratislava. The written informed consent was obtained from each participant before enrolment. The investigators preserved the full anonymity of all participants.

## Results

During the intervention in the LTCF, 78 subjects were screened for COVID-19 and 59 patients (47 women, 12 men) had positive pharyngeal swabs for SARS-CoV-2 RNA by RT-PCR and were diagnosed with Covid-19. Fifty-three patients (90%) had blood sampling during the initial screening phase. In 6 patients, blood sampling was not performed because of technical difficulties. These patients were excluded from the study. In 53 patients (11 men, 42 women) in which blood sampling was performed, the clinical variables and the results of biochemical and blood count analysis were available for evaluation and were included in the study. Seven patients (1 man, 6 women) were diagnosed with severe disease in initial triage and were immediately transferred to a hospital within the first 2 days of intervention. Remaining 45 patients (10 men, 36 women) remained under observation in LTCF and were screened for severe disease and triaged on a daily basis by the intervention team. Overall, 32 patients (6 men, 26 women) were admitted. Among these patients, 19 (2 men, 17 women) had hypoxemia required oxygen therapy, 16 patients (2 men, 14 women) were admitted to ICU, and 13 patients (2 men, 12 women) died. In 11 patients (1 man, 10 women) initially suffering from hypoxemia requiring oxygen therapy, the disease progressed, and they required intubation and mechanical ventilation. All these patients died because of severe ARDS. One male, initially suffering from hypoxemia requiring oxygen therapy died because of severe gastrointestinal bleeding from gastric adenocarcinoma. One female, initially suffering from hypoxemia requiring oxygen therapy suddenly died after short period of clinical improvement. An autopsy revealed pulmonary embolism. In hospitalized patients, various severe complications were observed. Six patients developed deep vein thrombosis, 6 developed congestive heart failure, 3 patients developed new atrial fibrillation and 5 developed acute renal failure. Baseline characteristics of patients are provided in Table 1. Major clinical findings of all enrolled patients are provided in supplementary table. The baseline serum concentrations of IL-6, CRP, procalcitonin, urea, creatinine, fibrinogen, AST, ALT, fasting glucose, total bilirubin and neutrophil count were significantly higher, and lymphocyte and eosinophil count was significantly lower in patients who developed hypoxemia. All patients included in our study seroconverted and developed IgG antibodies within 1 month of diagnosis. The median of baseline serum concentrations of D-dimer and ferritin and a median total count of leukocytes were not significantly different among groups of patients with and without hypoxemia requiring oxygen therapy. Patients who developed hypoxemia requiring oxygen therapy were also significantly more comorbid according to the higher median Charlson Comorbidity Index (Table 1). In the ROC analysis, IL-6 was identified as more robust marker of hypoxemia development than CRP, procalcitonin, CRP, fibrinogen, ALT, AST and total bilirubin and lymphocyte, neutrophil and eosinophil blood count. However, all of these variables were associated with hypoxemia (Table 2). The cut-off of 24 pg/mL for IL-6 showed the best combination of sensitivity and specificity. In the group of all screened LTCF residents, baseline IL-6 concentration > 24 pg/mL predicted the development of hypoxemia with a sensitivity of 88% and specificity of 89%. Positive predictive value (PPV) was 83%, and negative predictive value was (NPV) of 93%. After excluding the 7 patients diagnosed with severe Covid-19 and transferred to the hospital after the initial assessment, baseline IL-6 concentration over 24 pg/mL predicted the development of hypoxemia during the daily monitoring in the LTCF with the sensitivity of 100%, specificity 89, PPV of 77%, and NPV 100% (Table 3). Baseline CRP concentration with cut-off of 24 mg/L showed sensitivity and specificity inferior to IL-6 (Table 4). In multivariate analysis, baseline IL-6 concentration > 24 pg/mL was positively associated with the risk of hypoxemia development during follow up in LTCF residents after adjustment for CRP, age, gender, and glomerular filtration rate (Table 5).

**Table 1:** Baseline characteristics of patients. Variables are provided as median (25th percentile, 75th percentile)

	No hypoxemia requiring oxygen therapy (*n* = 26)	Hypoxemia requiring oxygen therapy (*n* = 19)	p (Mann-Whitney)	Cohen d
Age (years)	81 (73, 87)	87 (80.5, 90)	0.056	0.522
SpO2	0.96 (0.95, 0.97)	0.91 (0.86, 0.94)	< 0.0001	1.563
CCI	5 (4, 6)	7 (6, 8)	< 0.05	1.038
CRP (mg/L)	8.92 (3.223, 17.943)	70.69 (29.59, 142.46)	< 0.0001	1.398
IL-6 (pg/mL)	12.3 (7.3, 20.5)	43.1 (26.3, 116.7)	< 0.0001	1.880
D-dimer (mg/L)	1.215 (0.558, 2.625)	1.58 (0.78, 3.43)	0.198	0.366
Fibrinogen (g/L)	3.6 (3.3, 4.18)	4.45 (3.825, 5.825)	< 0.05	0.869
Procalcitonine (ng/mL)	0.02 (0.02, 0.03)	0.132 (0.048, 0.313)	< 0.0001	1.791
Ferritin (ug/L)	175.9 (94.13, 429.3)	295.18 (149.79, 778.02)	0.125	0.465
AST (ukat/L)	0.355 (0.29, 0.533)	0.755 (0.403, 1.12)	< 0.0001	1.214
ALT (ukat/L)	0.25 (0.16, 0.37)	0.35 (0.26, 0.54)	< 0.05	0.613
Sodium (mmol/L)	140.9 (137.45, 142.675)	139 (133.6, 145.6)	0.830	0.06
Potasium (mmol/L)	4.09 (3.85, 4.36)	3.71 (3.235, 4.503)	0.184	0.386
Glucose (mmol/L)	4.8 (4.2, 5.4)	5.9 (5.1, 7.1)	< 0.01	0.861
Urea (mmol/L)	6.2 (5.4, 7.5)	11 (6, 21.5)	< 0.01	0.794
Creatinine (umol/L)	74.5 (59.25, 101.25)	110.9 (73, 264)	< 0.05	0.729
Total bilirubin (umol/L)	8.75 (6.55, 11.75)	11.95 (9.8, 15.4)	< 0.0001	0.728
WBC (cells/mL)	4880 (4100, 6515)	5640 (4420, 8640)	0.167	0.395
LBC (cells/mL)	1565 (1045, 2083)	860 (580, 1300)	< 0.0001	1.035
NBC (cells/mL)	2680 (2015, 3800)	3890 (2950, 7450)	< 0.05	0.816
MBC (cells/mL)	490 (370, 615)	440 (250, 680)	0.712	0.107
EBC (cells/mL)	110 (30, 190)	10 (0, 30)	< 0.05	1.373
BBC (cells/mL)	20 (10, 37)	10 (10, 20)	0.155	0.398

*ALT* alanine aminotransferase, *AST* aspartate aminotransferase, *BBC* basophil blood count, *CCI* Charlson Comorbidity Index, *CRP* C-reactive protein, *EBC* eosinophil blood count, *IL-6* interleukin 6, *LBC* lymphocyte blood count, *MBC* monocyte blood count, *NBC* neutrophil blood count, *p* probability, *SpO2* oxygen saturation, *WBC* white blood cell blood count

**Table 2:** AUC and optimal cut-offs for evaluated markers of hypoxemia requiring oxygen therapy by receiver operating characteristic curve analysis

marker	AUC	95% CI of AUC	Asymptotic significance
IL-6	0.911	0.819–1	0.0001
CRP	0.887	0.777–0.996	0.0001
Fibrinogen	0.788	0.640–0.936	0.001
D-dimer	5.777	0.409–0.745	0.392
PCT	0.886	0.767–1	0.0001
Total bilirubin	0.712	0.535–0.888	0.036
Creatinine	0.644	0.443–0.846	0.152
AST	0.838	0.701–0.975	0.001
ALT	0.675	0.491–0.858	0.083
LBC	0.777	0.642–0. 912	0.001
NBC	0.728	0.582–0.783	0.007
EBC	0.841	0.730–0.953	0.0001

*ALT* alanine aminotransferase, *AST* aspartate aminotransferase, *AUC* area under curve, *95% CI* 95% confidence interval, *CRP* C-reactive protein, *EBC* eosinophil blood count, *IL-6* interleukin 6, *LBC* lymphocyte blood count, *NBC* neutrophil blood count, *PCT* procalcitonine, Asymptotic significance below 0.5 is regarded as statistically significant

**Table 3:** Test evaluation of baseline concentration of IL-6 > 24 pg/mL for predicting the development of hypoxemia requiring oxygen therapy during follow up after excluding patients admitted to hospital during initial triage

Statistic	Value	95% confidence interval
Sensitivity	100.00%	69.15–100.00%
Specificity	88.89%	70.84–97.65%
Disease prevalence	27.03%	13.79–44.12%
PPV	76.92%	53.42–90.64%
NPV	100.00%	Non available
Accuracy	91.89%	78.09–98.30%

*95% CI* 95% confidence interval, *NPV* negative predictive value, *PPV* positive predictive value

**Table 4:** Test evaluation of baseline concentration of CRP > 24 mg/L for predicting the development of hypoxemia requiring oxygen therapy during follow up after excluding patients admitted to hospital during initial triage

Statistic	Value	95% CI
Sensitivity	90.91%	58.72 to 99.77%
Specificity	79.41%	62.10 to 91.30%
Disease prevalence	24.44%	12.88 to 39.54%
PPV	58.82%	41.84 to 73.94%
NPV	96.43%	80.52 to 99.44%
Accuracy	82.22%	67.95 to 92.00%

*95% CI* 95% confidence interval, *NPV* negative predictive value, *PPV* positive predictive value

**Table 5:** Multivariate binary logistic regression analysis of the association of IL-6 > 24 pg/mL, CRP, glomerular filtration rate, gender, and age with the probability of development of hypoxemia requiring oxygen therapy during follow up

	p	OR	95% CI of OR
IL-6 > 24 pg/mL	< 0.05	39.741	1.838–859.426
CRP	0.203	1.028	0.985–1.072
age	0.187	1.133	0.943–1.364
gender (male)	0.175	0.088	0.003–2.944
GFR	0.773	0.979	0.850–1.128

*CRP* C-reactive protein, *GFR* glomerular filtration rate, *95% CI* 95% confidence interval, *IL-6* interleukin 6, *OR* odds ratio, *p* probability

## Discussion

Our retrospective data analysis evaluated the potential role of biomarkers of inflammation, IL-6, CRP, procalcitonin, D-dimer and fibrinogen in the prediction of severe disease and need for oxygen therapy in residents of LTCF during Covid-19 outbreak. It suggests that IL-6 is a most robust predictor of hypoxemia requiring oxygen therapy. The concentration of IL-6 > 24 pg/mL at the initial assessment is showing the best combination of sensitivity and specificity in predicting the hypoxemia requiring oxygen therapy.

There is a substantial body of evidence linking the IL-6 concentration to the severity of disease and unfavorable outcome of Covid-19 [13, 14, 16–18, 20–23]. However, to our knowledge, this study is the first study which evaluated the ability of IL-6 to predict the need for supplementary oxygen administration as a surrogate marker of severe Covid-19 requiring hospital admission in the population of high risk elderly patients. It also suggests clinical implications for intervention procedures for Covid-19 outbreak in LTCF. Han et al. examined the predictive value of various cytokines and concluded that IL-6 is the best predictor of severe Covid-19 [14]. A metanalysis of 9 studies concluded that increased IL-6 is highly associated with severe disease. In this study, patients with severe Covid-19 had mean IL-6 58 pg/mL compared to 17 pg/mL in mild disease [22]. A study by Herold et al. found that IL-6 > 80 pg/mL the predicts respiratory failure and need for mechanical ventilation in Covid-19. Like in our study, IL-6 was superior to CRP in ROC analysis. Chen et al. found cut-off 80 pg/mL of IL-6 differentiates the survivors from the non-survivors [23]. We suggest that we found much lower optimal cut-off value (24 pg/mL compared to 80 pg/mL) because we choose less severe endpoint (need for supplementary oxygen). Overall, we can conclude that our study is in concordance with previous studies and adds new aspects to the known body of evidence.

### Clinical significance and implications

The residents of LTCF are one of the most vulnerable populations of the Covid-19 pandemic [8–10]. The outbreak of Covid-19 in LTCF might significantly burden the local health care system. Timely and effective intervention is essential to reduce morbidity and mortality during such outbreak. According to the proposed guideline by Kim et al., the first phase of response should include broad testing a quick identification of cases and their clinical assessment and triage. In the next phase, monitoring of patients should be implemented in order to quickly identify the patients in need of hospital care [11]. Identification of patients at high risk of deterioration during the initial assessment may significantly improve the monitoring process by allocating the resources to high-risk patients more effectively. Especially in the case of limited human resources, the focus on high-risk patients is might contribute to mortality reduction and improve the overall outcome of the outbreak.

In our study, all patients that developed hypoxemia requiring oxygen therapy during the follow up had the baseline concentration of IL-6 over 24 pg/mL. As a screening tool, it provides excellent sensitivity with an acceptable specificity over 88%. It may be used to identify the patients with a high risk of hypoxemia. It also identifies the patients with low risk for hypoxemia with great negative predictive value. This might help in the decision making for admission during the initial triage. Baseline CRP concentration with cut-off of 24 mg/L showed sensitivity and especially specificity inferior to IL-6, however, might be used as cheaper alternative in low resource setting.

For the purposes of our study, we defined that the residents that developed hypoxemia requiring oxygen therapy are the cases that needed close monitoring and early transfer to the hospital. The rationale is that patients who developed hypoxemia requiring oxygen therapy are those that might benefit from the early pharmacological treatment in order to reduce mortality. According to the body of evidence on remdesivir and dexamethasone, these drugs significantly improves the outcome in the patients needing conventional oxygen therapy [24, 25]. Thus, the patients with early disease but in the high risk of development of hypoxemia are those patients who will profit from close observation and rapid initiation of remdesivir and dexamethasone in case of progression of Covid-19.

### IL-6 and development of severe Covid-19

IL-6 is produced by stromal cells and virtually all immune system cells in the lungs and its secretion is stimulated by proinflammatory cytokines, especially interleukin 1β (IL-1β) and tumor necrosis factor α (TNFα). In the early stages of the infection, it is produced by lung macrophages after stimulation of toll-like receptors [26]. An important trait of IL-6 upregulation in Covid-19 is that it precedes the development of acute lung injury that implicates its usability as an early marker of severe disease [22]. However, there is controversy if excessive IL-6 synthesis is true a cornerstone of the pathogenesis of respiratory failure in Covid-19 or is just an epiphenomenon of increased IL-1β and TNFα in the cytokine storm [16]. Predominant theory is that overexpression of IL-6 have a crucial role in the incitement and propagation of the so-called cytokine storm leading to lung injury and ARDS [16, 17]. It is believed that IL-6 increases the permeability of lung capillaries driving the ARDS development and also stimulates the coagulation pathway leading to microthrombi in lung circulation and increases the risk of thrombotic event [26]. A study by Giamarellos-Bourboulis et al. suggests that patients with severe respiratory failure in Covid-19 suffer from distinct types of immune dysregulation which are mediated by IL-6 upregulation. This dysregulation is characterized by high production of proinflammatory cytokines by monocytes and macrophages and CD4 lymphocyte depletion that contributes to the progression of inflammation of lung parenchyma [17]. The direct role of IL-6 in Covid-19 pathogenesis is further supported by findings that IL-6 inhibition improves the prognosis of severe Covid-19 [26, 27].

### Other markers of severe disease

In our study, patients who developed hypoxemia had significantly higher serum concentrations of AST, ALT, CRP, serum glucose, creatinine, procalcitonin, and fibrinogen. They also had significantly higher blood count of neutrophils and lower count of lymphocytes and eosinophils. These variables were identified as markers of severe disease by the previous studies [13]. Medians of other well-established markers of serious disease, D-dimer and ferritin were higher in patients who developed hypoxemia, however, the differences were not statistically significant. Using ROC analysis, we concluded that IL-6 is better marker of hypoxemia than CRP or other evaluated variables. Other proinflammatory cytokines, like IL-1β or TNF-α are also associated with severity of Covid-19 and might also serve as biomarkers [28, 29]. However, because the primary goal of our study was to improve the practical algorithm for intervention in LTCF, we focused on examination of biomarkers that are well established in clinical practice and are available for evaluation in most commercial biochemical laboratories in Slovakia.

### Prognosis of LTCF residents suffering from Covid-19

According to an epidemiologic study by McMichael et al., during the outbreak in one LTCF in Washington, USA, 54.5% of residents required hospitalization and required hospital admission and 33.7% of infected residents died [8]. In our study, we identified 59 residents with positive swabs for SARS-CoV-2. Of these patients. Thirty-two patients (54%) were admitted to hospital which is similar to the study by McMichael et al. In our study, 13 patients (22%) died. That is less than in the study by McMichael et al., however, this difference of proportion might be attributed to potentially different age and comorbidity status of residents.

### Limitations

The limitations of our study are the relatively low sample size and retrospective design. Larger prospective studies are needed to obtain more robust data and to evaluate if the examination of IL-6 during the initial assessment leads to better prognosis of LTCF residents and improves the management of the Covid-19 outbreaks in the LTCFs.

Because of the retrospective design of the study, there might be a concern of bias in the sensitivity of the diagnosis of hypoxemia between groups of hospitalized and outpatient residents. The hospitalized patients were naturally more closely monitored and therefore might be more likely to be diagnosed with hypoxemia. However, we regard this potential bias as insignificant because the outpatient residents were daily monitored for the symptoms and signs of respiratory failure, and patients suffering from dyspnea and patients with tachypnea and/or SpO2 below 90% were transferred to hospital. In normoxic patients, the bias of pulse oximetry comparing to SaO2 is regarding to be insignificant. It reliably identifies the patients with SaO2 below 90% and is a reliable screening tool for hypoxemia with very high negative predictive value [30].

Another limitation is that we did not evaluate the IL-1β as a possible biomarker of hypoxemia. IL-1β is believed to contribute to the cytokine storm in Covid-19. It is the cytokine which stimulates the IL-6 production and therefore is on the level above the IL-6 in the cascade of cytokine storm [28]. The theory that IL-1β have an important role in the pathogenesis of Covid-19 is widely accepted [29]. However, recent study by Mandel et al. found that baseline concentration of IL-1β is not significantly higher in non-survivors [18]. Giamarellos-Bourboulis et al. concluded that immune dysregulation in severe Covid-19 is directly driven by IL-6, not IL-1β upregulation [17].

Our study included more females than males. This proportion reflects the LTCF population in which female to male ratio was three to one and is not a result of deliberate selection.

## Conclusions

Baseline IL-6 concentrations over 24 pg/mL in LTCF residents suffering from Covid-19 predicts the development of hypoxemia requiring oxygen therapy with excellent sensitivity and good specificity. Patients with the IL-6 above 24 pg/m at initial assessment seems to be at high risk of development of respiratory failure and might benefit from early hospitalization and close follow-up. Further studies are needed to evaluate the real benefits of systematic examination of IL-6 during the initial assessment and its use as predictive factor of severe disease.

## Supplementary Information

**Additional file 1: Supplementary table**: Clinical characteristics, major comorbidities, major complications and most significant biochemical and blood count variables provided for individual patients.

## Data Availability

The dataset used and analyzed during this study is available as a supplementary material.

## Abbreviations

ALT: Alanine aminotransferase
ARDS: Acute respiratory distress syndrome
AST: Aspartate aminotransferase
AUC: Area under curve
BBC: Basophil blood count
CCI: Charlson Comorbidity Index
Covid-19: Coronavirus disease 2019
EBC: Eosinophil blood count
IL-6: Interleukin 6
IL-1β: Interleukin 1β
LBC: Lymphocyte blood count
LTCF: Long term care facility
MBC: Monocyte blood count
NBC: Neutrophil blood count
NPV: Negative predictive value
p: probability
PPV: Positive predictive value
ROC: Receiver operating characteristic curve
SARS-CoV-2: Severe acute respiratory distress syndrome coronavirus 2
SaO2: Oxygen saturation from arterial blood
SpO2: Oxygen saturation by pulse oximetry
TNF-α: Tumor necrosis factor α
WBC: White blood cell blood count
