# Supplementary material for: Role of interleukin 6 as a predictive factor for a severe course of Covid-19: retrospective data analysis of patients from a long-term care facility during Covid-19 outbreak
Source: BMC Infect Dis. 2021 Mar 29;21:308. doi: 10.1186/s12879-021-05945-8 (PMC8006112; doi:10.1186/s12879-021-05945-8)
Supplement: Supplementary file 1 — Additional file 1: Supplementary table: Clinical characteristics, major comorbidities, major complications and most significant biochemical and blood count variables provided for individual patients. [file 12879_2021_5945_MOESM1_ESM.docx]

| Subject | Age range | Sex | Major comorbidity | Admission to hospital | SpO2 at initial assessment | Hypoxia requiring oxygen support | Admission to ICU | Mechanical ventilation | Major complications | CRP (mg/L) | IL-6 (pg/mL) | PCT (ng/mL) | WBC  (x1000 cells/mL) | LBC (x1000 cells/mL) |
| --- | --- | --- | --- | --- | --- | --- | --- | --- | --- | --- | --- | --- | --- | --- |
| 1 | 80 - 89 | male | Ca | yes | 0.93 | yes | no | no | Hematemesis | 103.98 | 198.3 | 0.13 | 4.89 | 0.86 |
| 2 | 80 - 89 | female | Stroke, AH, CKD | yes | 0.93 | yes | yes | yes | ARDS, HF, RF | 88.03 | 160.1 | 0.07 | 2.07 | 0.58 |
| 3 | 90 - 99 | female | DM, HF, CKD | yes | 0.86 | yes | yes | no | UTI | 265.97 | 137.9 | 0.17 | 12.51 | 1.56 |
| 4 | 80 - 89 | female | AH, HF, COPD | yes | 0.97 | yes | yes | no | no | 29.59 | 125.4 | 0.02 | 3.69 | 0.76 |
| 5 | 80 - 89 | female | AH, HF, CKD | yes | 0.93 | yes | yes | yes | ARDS, HF, DVT | 74.02 | 108 | 0.05 | 5.4 | 1.1 |
| 6 | 90 - 99 | female | AH | yes | 0.92 | yes | yes | yes | ARDS, DVT | 232.52 | 100.4 | 0.136 | 6.83 | 0.88 |
| 7 | 90 - 99 | female | Stroke, AH, HF, CKD | yes | 0.80 | yes | yes | yes | ARDS, Shock | 142.46 | 50 | 0.13 | 7.78 | 0.29 |
| 8 | 80 - 89 | female | Stroke, AH, DM, HF, MI, CKD | yes | 0.95 | yes | yes | yes | ARDS, HF, RF, Hypernatremia | 70.69 | 43.3 | 2.55 | 6.71 | 1.56 |
| 9 | 80 - 89 | female | Stroke, AH | yes | 0.94 | yes | yes | no | Sepsis | 130.57 | 43.1 | 0.05 | 3.81 | 1.06 |
| 10 | 70 – 79 | female | AH | no | 0.93 | no | no | no | no | 36.87 | 42.6 | 0.02 | 6.44 | 1.62 |
| 11 | 80 – 89 | female | AH | no | 0.95 | no | no | no | no | 18.78 | 41.7 | 0.02 | 2.18 | 0.66 |
| 12 | 70 – 79 | female | AH, MI | yes | 0.93 | yes | no | no | AF | 43.71 | 37.9 | 0.38 | 5.86 | 0.63 |
| 13 | 80 – 89 | female | AH, DM | yes | 0.70 | yes | yes | yes | ARDS | 253.79 | 37.4 | 0.68 | 10.9 | 0.34 |
| 14 | 90 – 99 | female | AH, DM, HF | yes | 0.89 | yes | no | no | DVT | 40.69 | 28.5 | 0.04 | 4.42 | 1.21 |
| 15 | 80 – 89 | female | Stroke, AH, HF, CKD | yes | 0.79 | yes | yes | no | RF | 66.91 | 27.3 | 0.29 | 8.64 | 0.49 |
| 16 | 90 – 99 | female | Stroke, AH, MI, HF, CKD | yes | 0.94 | yes | no | no | no | 23,92 | 25,3 |  | 5,04 | 1,3 |
| 17 | 80 – 89 | male | AH, CKD | no | 0.95 | no | no | no | no | 9,27 | 24,9 | 0,02 | 4,09 | 0,77 |
| 18 | 70 - 79 | female | AH, CKD | yes | 0.97 | yes | no | no | DVT, PE | 9,52 | 24,6 | 0,03 | 5,64 | 2,36 |
| 19 | 80 – 89 | female | AH | no | 0.97 | no | no | no | no | 5,37 | 21,6 | 0,02 | 4,51 | 1,71 |
| 20 | 90 – 99 | male | AH, DM, HF, CKD | yes | 0.97 | no | no | no | no | 15,43 | 21,3 | 0,06 | 1,95 | 0,92 |
| 21 | 80 – 89 | female | AH, DM, HF | yes | 0.94 | no | no | no | no | 11,08 | 21,1 | 0,02 | 4,91 | 2,62 |
| 22 | 90 – 99 | female | AH, HF | no | 0.96 | no | no | no | no | 5,42 | 20,5 | 0,02 | 4,63 | 0,92 |
| 23 | 80 - 89 | female | AH, DM | no | 0.97 | no | no | no | no | 38,02 | 18,3 | 0,02 | 6,91 | 2,33 |
| 24 | 80 - 89 | female | Stroke, AH, DM | yes | 0.96 | no | no | no | Severe hyponatremia | 10,77 | 18,2 | 0,02 | 4,25 | 1,03 |
| 25 | 60 – 69 | female | AH | yes | 0.94 | no | no | no | no | 11 | 15,6 | 0,02 | 3,22 | 1,41 |
| 26 | 60 – 69 | male |  | yes | 0.94 | no | no | no | no | 10,01 | 15,4 | 0,05 | 5,58 | 1,16 |
| 27 | 80 - 89 | male | AH | no | 0.96 | no | no | no | no | 5,79 | 14,3 | 0,03 | 4 | 1,09 |
| 28 | 60 - 69 | male | AH, CKD | no | 0.97 | no | no | no | no | 5,07 | 13 | 0,07 | 4,8 | 1,58 |
| 29 | 80 – 89 | female | AH | yes | 0.88 | yes | yes | yes | ARDS, HF, AF | 3,03 | 12,9 | 0,03 | 5,16 | 0,77 |
| 30 | 90 – 99 | female | Stroke, AH, DM, HF | yes | 0.92 | no | no | no | HF, AF | 57,37 | 12,3 | 0,03 | 10,06 | 1,62 |
| 31 | 80 – 89 | female | AH | yes | 0.97 | no | no | no | RF | 8,57 | 12,1 | 0,03 | 5,98 | 1,51 |
| 32 | 90 – 99 | female | AH, MI, HF, CKD | yes | 0.89 | yes | no | yes | ARDS, DVT | 6,47 | 11,51 | 0,134 | 11 | 1,99 |
| 33 | 90 – 99 | female | AH, DM, HF | no | 0.99 | no | no | no | no | 2,55 | 10 | 0,02 | 6,54 | 2,85 |
| 34 | 80 - 89 | male | AH, COPD | no | 0.95 | no | no | no | no | 3,44 | 10 | 0,03 | 4,13 | 0,95 |
| 35 | 70 – 79 | male | AH | yes | 0.94 | no | no | no | no | 11,38 | 9,9 | 0,03 | 6,06 | 2,21 |
| 36 | 60 – 69 | male | AH, DM | no | 0.99 | no | no | no | no | 1 | 8 | 0,02 | 5,1 | 1,81 |
| 37 | 80 - 89 | female | AH, DM | no | 0.96 | no | no | no | no | 2,05 | 7,4 | 0,02 | 5,45 | 1,91 |
| 38 | 80 - 89 | female | Stroke, AH, DM | no | 0.97 | no | no | no | no | 1 | 7,3 | 0,02 | 3,61 | 1,55 |
| 39 | 60 – 69 | female |  | no | 0.96 | no | no | no | no | 1,83 | 7,1 | 0,02 | 3,82 | 1,46 |
| 40 | 80 - 89 | female | AH | no | 0.96 | no | no | no | no | 12,5 | 7 | 0,02 | 3,31 | 0,84 |
| 41 | 80 - 89 | female | AH | no | 0.96 | no | no | no | no | 2,18 | 5,5 | 0,02 | 7 | 2,11 |
| 42 | 70 – 79 | female | AH | no | 0.96 | no | no | no | no | 5,05 | 5,4 | 0,03 | 4,85 | 2,17 |
| 43 | 50 – 59 | female |  | no | 0.98 | no | no | no | no | 1,18 | 4,9 | 0,02 | 10,83 | 5,2 |
| 44 | 70 – 79 | female | Stroke | no | 0.98 | no | no | no | no | 4,05 | 4,6 | 0,02 | 6,55 | 2,44 |
| 45 | 80 – 89 | female | AH, MI, HF, CKD | yes | 0.90 | yes | yes | yes | ARDS, Shock, DVT | 60,89 |  | 0,14 | 11,02 | 0,81 |
| 46 | 50 – 59 | female | COPD | yes | 0.92 | no | no | no | UTI | 90,59 |  |  | 7,58 | 1,35 |
| 47 | 60 – 69 | female | Stroke, CKD | yes | 0.81 | yes | yes | yes | ARDS, Shock | 578,41 |  | 48,61 | 4,3 | 0,4 |
| 48 | 80 – 89 | male | AH, DM, CKD | yes | 0.97 | no | no | no | no | 35,12 |  | 0,197 | 4,71 | 1,74 |
| 49 | 70 – 79 | female | AH | yes | 0.94 | no | no | no | UTI | 94,27 |  | 0,02 | 4,81 | 1,46 |
| 50 | 80 – 89 | male | Stroke, AH, HF, Ca | yes | 0.95 | no | yes | yes | ARDS, RF, HF | 99,65 |  |  | 11,59 | 0,7 |
| 51 | 80 – 89 | female | Stroke, AH, DM | no | 0.93 | no | no | no | no | 3,15 |  |  | 6,28 | 2 |
| 52 | 80 – 89 | female | AH | no | 0.95 | no | no | no | no | 22,06 |  |  | 8,45 | 3,27 |
| 53 | 70 – 79 | female | AH | no | 0.94 | no | no | no | no | 2,16 |  |  | 5,69 | 1,9 |

**Supplementary table**: Clinical characteristics, major comorbidities, major complications and most significant biochemical and blood count variables provided for individual patients.

AH: arterial hypertension, AF: atrial fibrilation, ARDS: acute respirotory distress syndrome, Ca: carcinoma, CRP: C-reactive protein, COPD: chronic obstructive pulmonary disease, CKD: chronic kidney disease, DM: diabetes mellitus, DVT: deep vein trombosis, ICU: intensive care unit, HF: heart failure, LBC: lymphocyte blood count, MI: myocardial infarction, PE: pulmonary embolism PCT: procalcitonine, WBC: white cell blood count
